# Supplementary figures and images for: Genetic Variance Estimation over Time in Broiler Breeding Programmes for Growth and Reproductive Traits
Source: Animals (Basel). 2023 Oct 24;13(21):3306. doi: 10.3390/ani13213306 (PMC10649193; doi:10.3390/ani13213306)

(a)

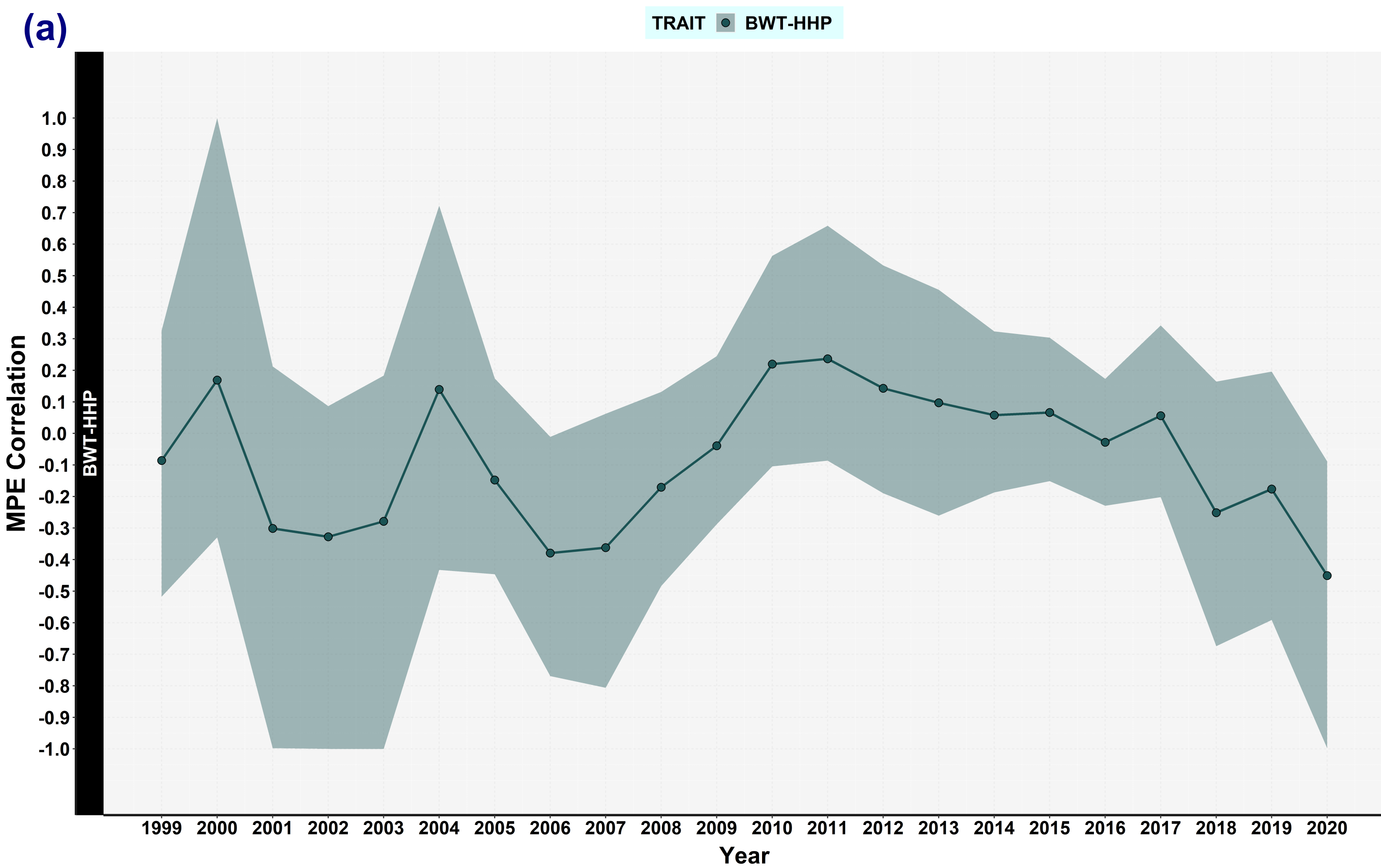

(b)

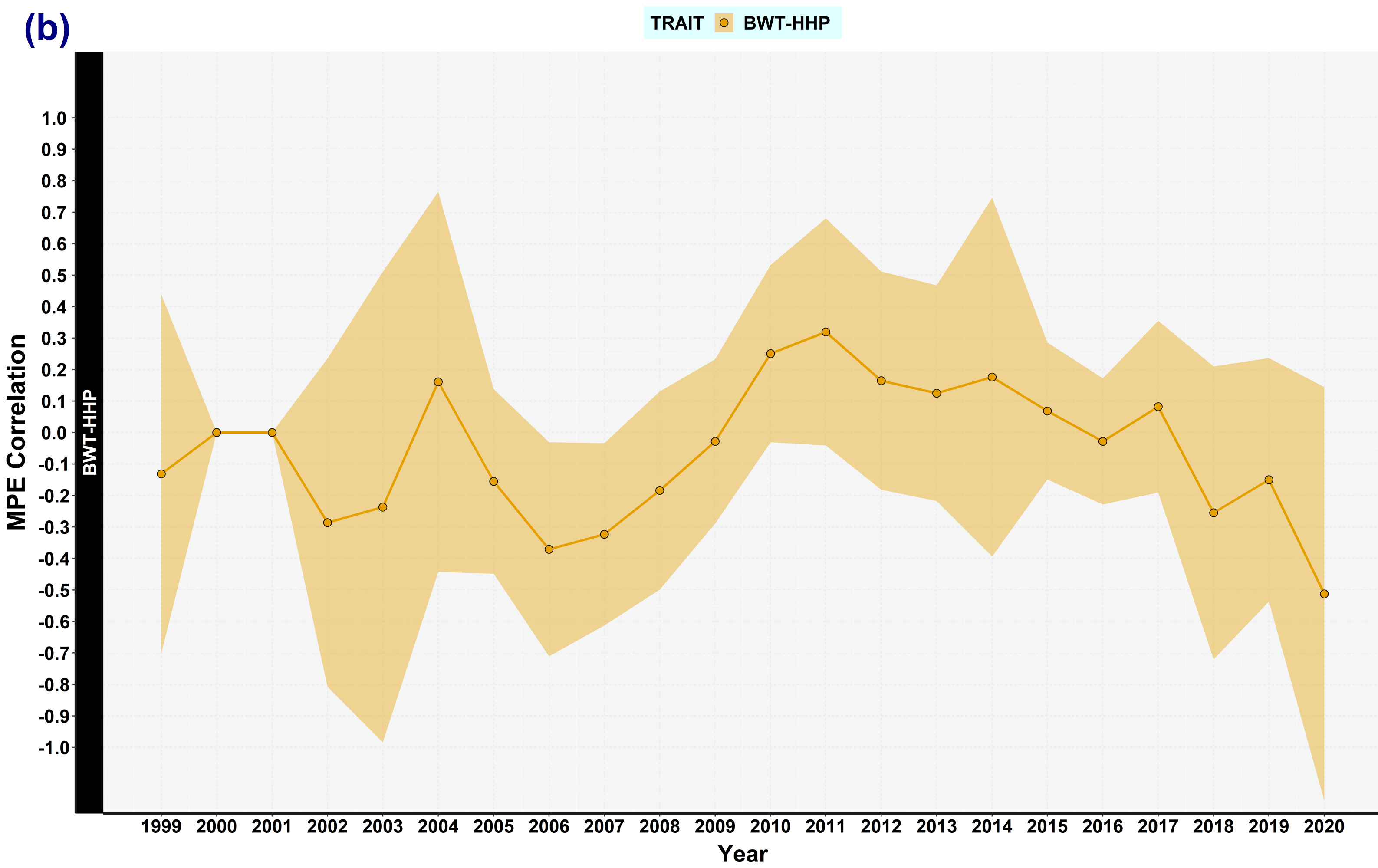

Supplement: Supplementary file 1 [file animals-13-03306-s001.zip › NEW_Figure_#S1_MPE_correlation.pdf]

**(a)**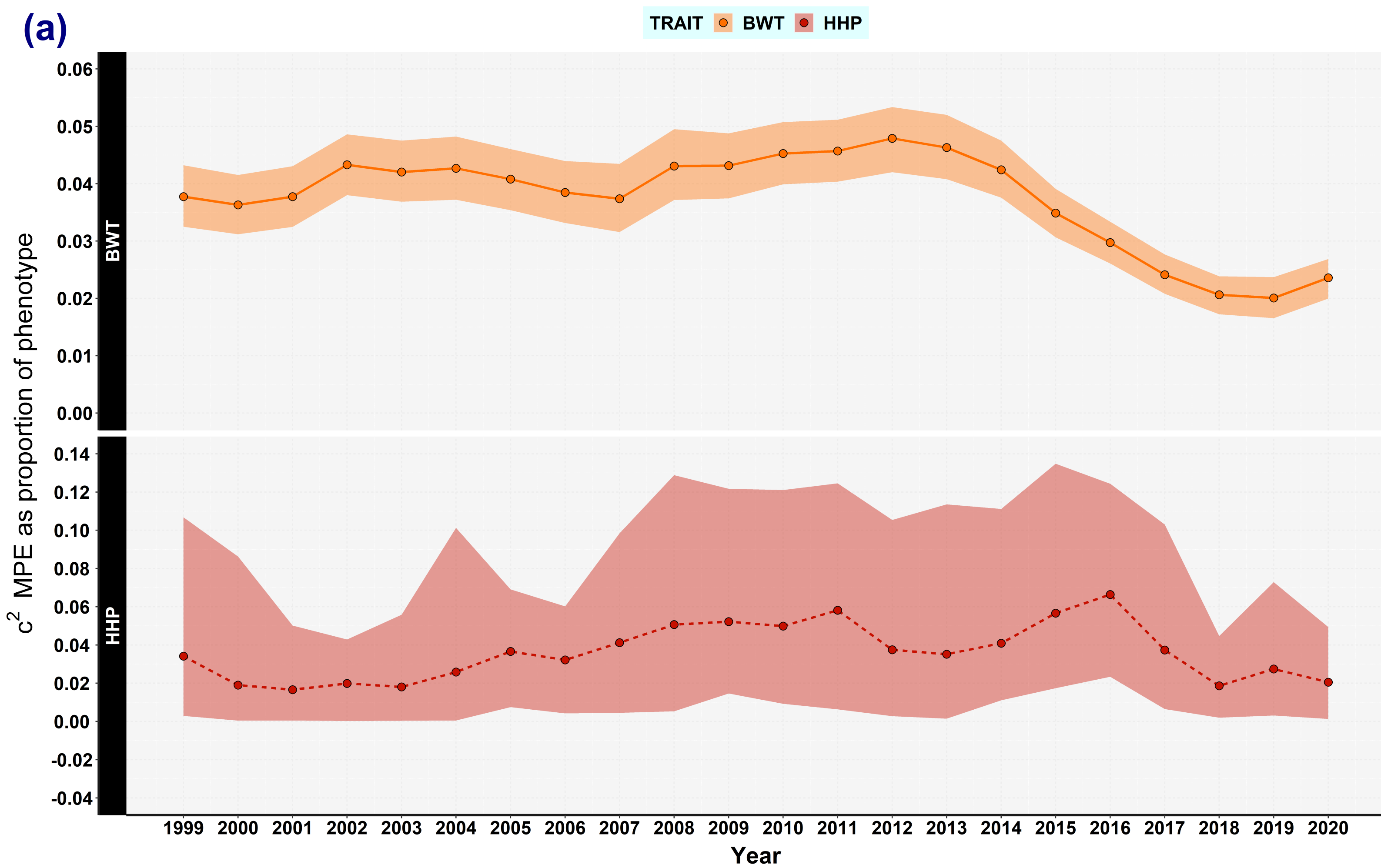**(b)**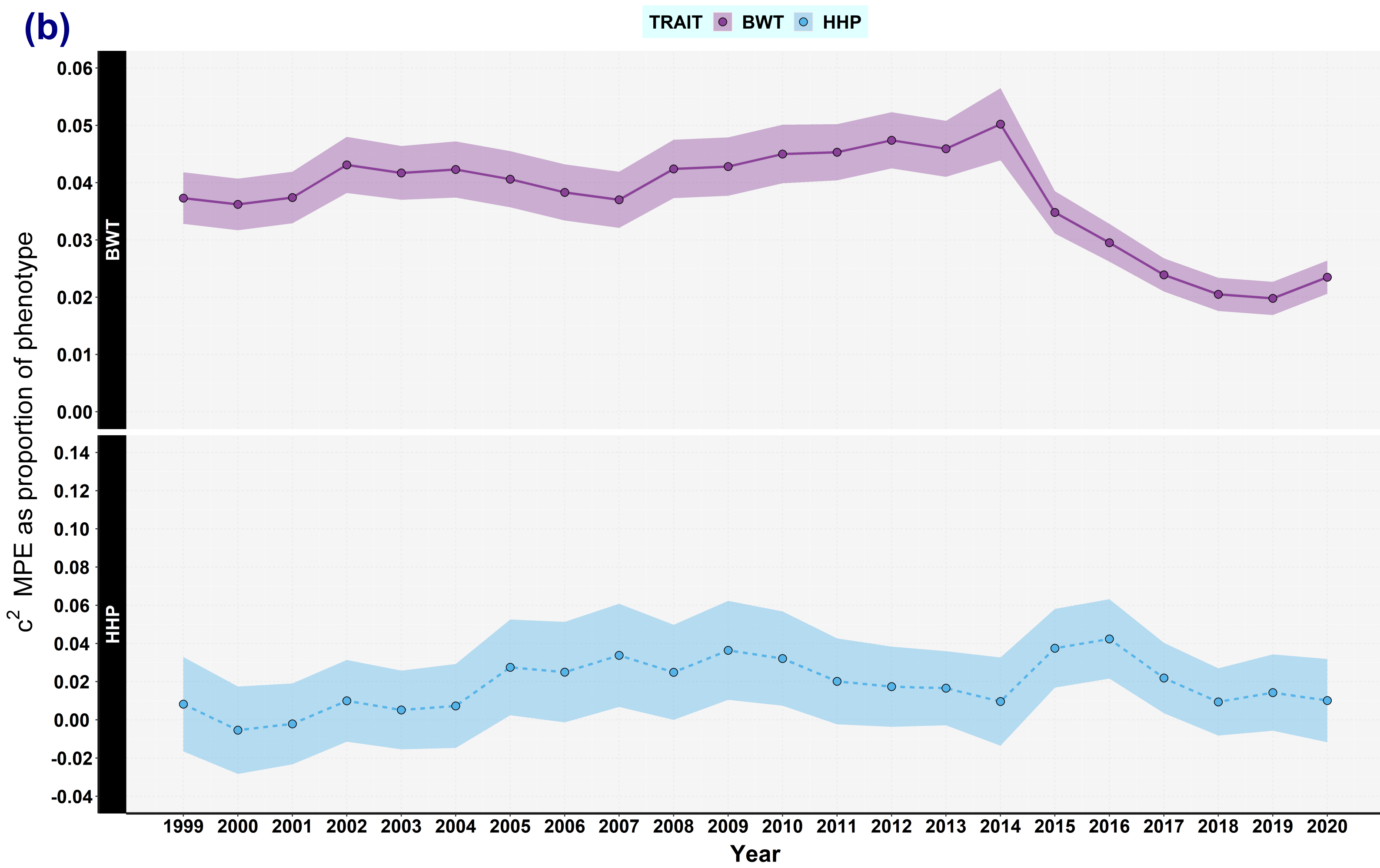

Supplement: Supplementary file 1 [file animals-13-03306-s001.zip › NEW_Figure_#S2_c2_MPE_in_proporction_of_phenotype.pdf]

TRAIT   BWT   BWT-HHP   HHP

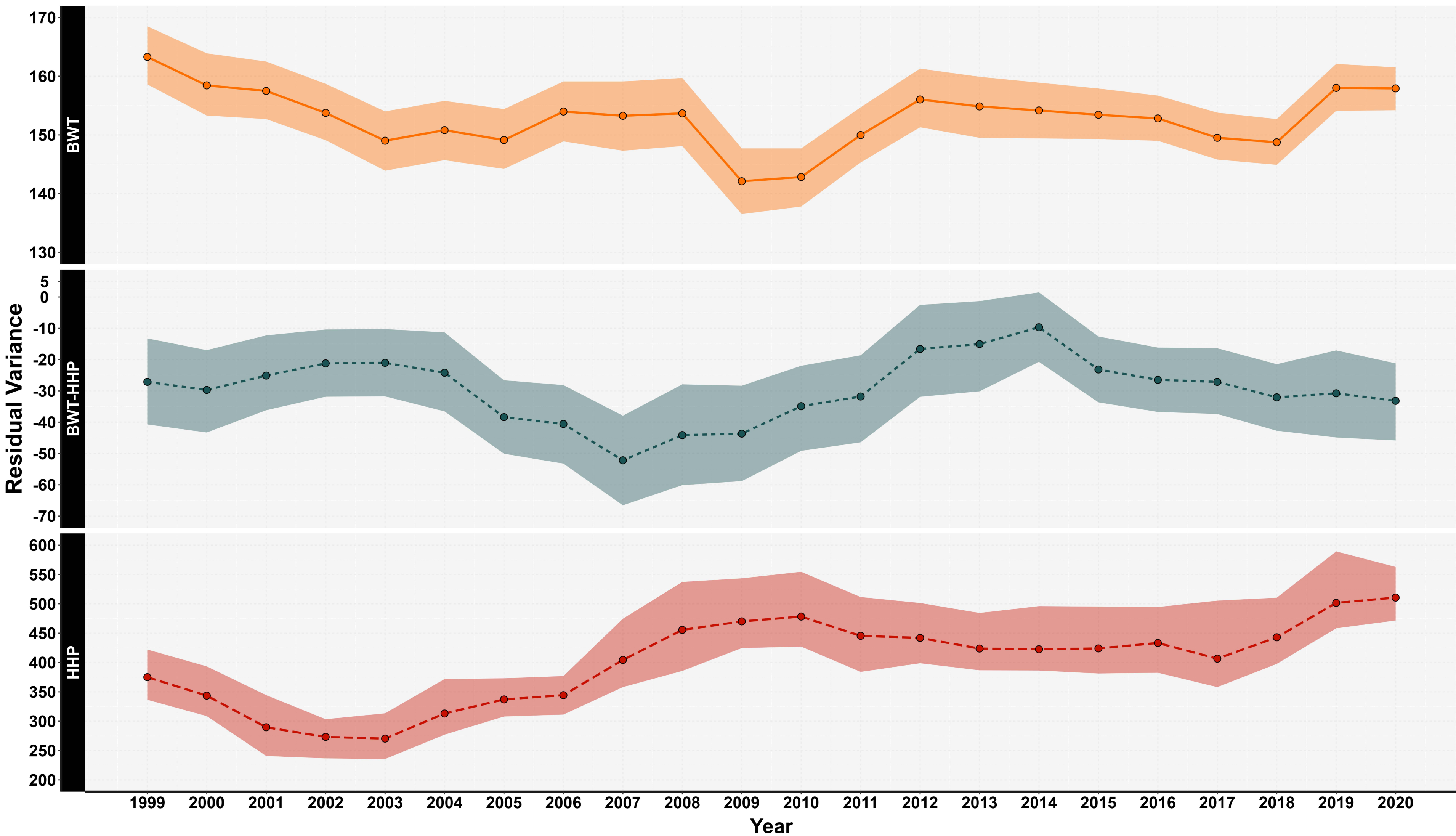

Supplement: Supplementary file 1 [file animals-13-03306-s001.zip › NEW_Figure_#S3_Residual_variance_Gibbs.pdf]

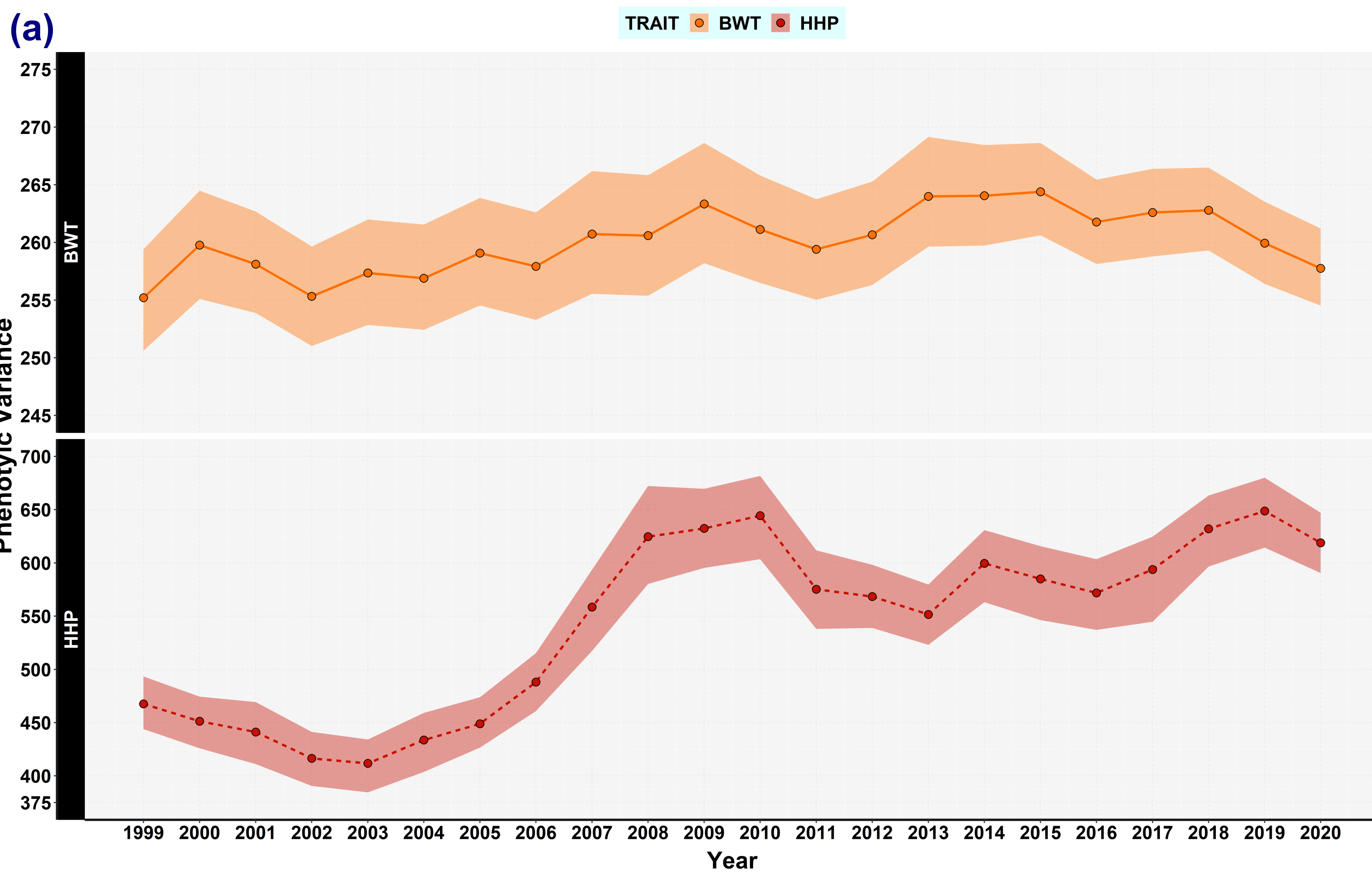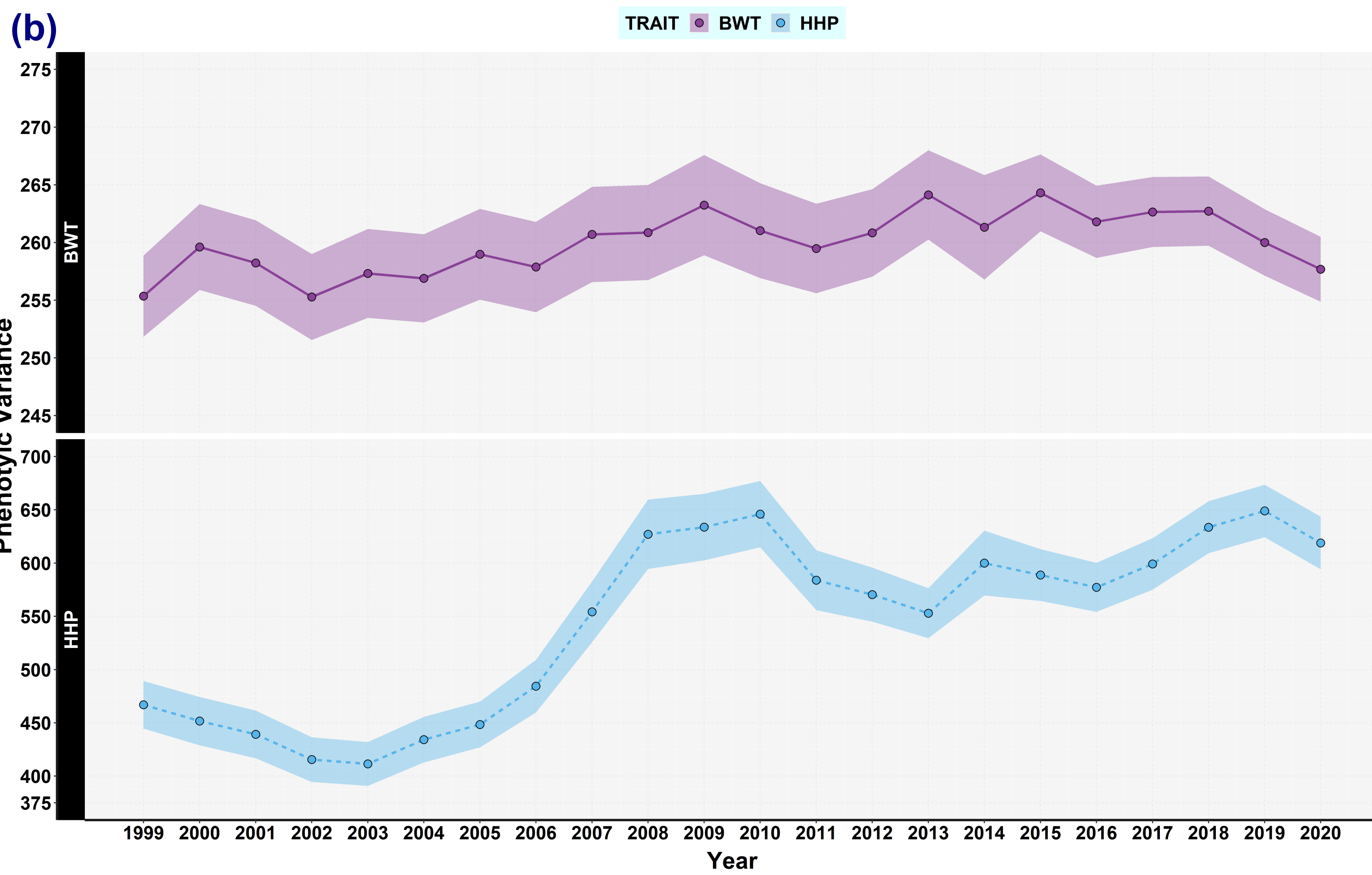

Supplement: Supplementary file 1 [file animals-13-03306-s001.zip › NEW_Figure_#S4_Phenotypic_variance.pdf]
